# French Criticism on Our Specialists

**Published:** 1878-04

**Authors:** 


French Criticism of Our Specialists.—Formerly the object was to prevent specialists in medicine, but times have changed greatly since then. The Academy and Faculty have abolished the kind of ostracism, whose victims they were for a long time. But we have not progressed so far as some foreign countries.

We find, in looking over the American journals, the announcements of certain faculties where all the chairs are occupied by specialists, whose enumeration is often curious. Thus, there is a professor of the anatomy of the ear, a professor of the physiology of heart diseases, etc. We do not find the name of a single professor of anatomy or pathology.

But there are, let it be remarked, besides these interlopers, good schools, whose professors are in no respect second to those of old Europe.—*La France Medicale.*

